# Origin of Pressure-induced Superconducting Phase in K_*x*_Fe_2−*y*_Se_2_ studied by Synchrotron X-ray Diffraction and Spectroscopy

**DOI:** 10.1038/srep30946

**Published:** 2016-08-08

**Authors:** Yoshiya Yamamoto, Hitoshi Yamaoka, Masashi Tanaka, Hiroyuki Okazaki, Toshinori Ozaki, Yoshihiko Takano, Jung-Fu Lin, Hidenori Fujita, Tomoko Kagayama, Katsuya Shimizu, Nozomu Hiraoka, Hirofumi Ishii, Yen-Fa Liao, Ku-Ding Tsuei, Jun’ichiro Mizuki

**Affiliations:** 1 Graduate School of Science and Technology, Kwansei Gakuin University, 2-1 Gakuen, Sanda, Hyogo 669-1337, Japan; 2 RIKEN SPring-8 Center, RIKEN, 1-1-1 Kouto, Mikazuki, Sayo, Hyogo 679-5148, Japan; 3 MANA, National Institute for Materials Science, 1-2-1 Sengen, Tsukuba, Ibaraki 305-0047, Japan; 4 Advanced Institute for Materials Research, Tohoku University, 2-1-1 Katahira, Aoba, Sendai, Miyagi 980-8577, Japan; 5 Department of Geological Sciences, The University of Texas at Austin, Austin, Texas 78712, USA; 6 Center for High Pressure Science and Technology Advanced Research (HPSTAR), Shanghai 201203, China; 7 Center for Science and Technology under Extreme Conditions (KYOKUGEN), Graduate School of Engineering Science, Osaka University, Toyonaka, Osaka 560-8531, Japan; 8 National Synchrotron Radiation Research Center, Hsinchu 30076, Taiwan

## Abstract

Pressure dependence of the electronic and crystal structures of K_*x*_Fe_2−*y*_Se_2_, which has pressure-induced two superconducting domes of SC I and SC II, was investigated by x-ray emission spectroscopy and diffraction. X-ray diffraction data show that compressibility along the *c*-axis changes around 12 GPa, where a new superconducting phase of SC II appears. This suggests a possible tetragonal to collapsed tetragonal phase transition. X-ray emission spectroscopy data also shows the change in the electronic structure around 12 GPa. These results can be explained by the scenario that the two SC domes under pressure originate from the change of Fermi surface topology. Our results here show the pronounced increase of the density of states near the Fermi surface under pressure with a structural phase transition, which can help address our fundamental understanding for the appearance of the SC II phase.

Since the discovery of high-temperature superconductivity in F-doped LaFeAsO in 2008^1^, many iron-based superconductors with different crystal structures have been synthesized and are still hot topics in condensed matter physics. Most iron-superconductor families have FeAs or FeSe planes as the common layers, which correlate to the superconductivity. The crystal structure of FeSe is the simplest of these iron-based superconductors with *T*_c_ = 8 K^2^. Moreover, it was recently found that a single FeSe layer on SrTiO_3_ showed high *T*_c_ of 65–100 K^3^,^4^.

Intercalation to FeSe layers by alkaline atoms also raised *T*_c_ to 30–46 K in bulk iron-based superconductors of *A*_*x*_Fe_2−*y*_Se_2_ (*A* = K, Rb, Cs)^5^,^6^,^7^,^8^. Therefore, in these systems electron-doping to the FeSe layer may play an important role in superconductivity. The electron-doping causes a Fe-deficiency of the FeSe layer to keep the charge valance, and this system is called 122* phase. These new iron-defected systems (122* family) have attracted many interests because of the following several unique features, which are very different from other iron-based superconductors^8^. (i) This system shows intrinsic phase separation^9^. It consists of 122-type superconductor KFe_2_Se_2_ and 245-type AFM insulator K_2_Fe_4_Se_5_ with 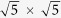 vacancy order which disappears around 10 GPa^10^,^11^,^12^,^13^,^14^,^15^. (ii) They have an unprecedented high Néel temperature of 559 K and large magnetic moment of ~3.3 *μ*_B_^16^,^17^. This magnetic moment is the largest among pnictide and chalcogenide iron-superconductors. (iii) Unlike the usual iron-based superconductor, there are no hole pockets at Fermi surface which enhances the Fermi surface nesting^18^. (iv) *T*_c_ of *A*_*x*_Fe_2−*y*_Se_2_ gradually drops with pressure, and superconductivity (SC I) disappears around 10 GPa. However, interestingly, further pressure induces a new superconductivity (SC II) suddenly around 11 GPa. The SC II phase shows higher *T*_c_ than the SC I phase^19^,^20^.

Recently, single phase non-superconducting K_2_Fe_4_Se_5_ was synthesized, and the pressure-temperature phase diagram was revealed^20^. By comparing the K_2_Fe_4_Se_5_ and K_*x*_Fe_2−*y*_Se_2_ phase diagrams, the phase separation in the SC II region was suggested, and the superconducting phase attributed to the 122 phase. Therefore, this means that superconducting phase with KFe_2_Se_2_ and non-superconducting phase with K_2_Fe_4_Se_5_ co-exist in the SC II phase.

A theoretical study of the SC I and SC II phases in the 122* system suggested that superconducting symmetry is *d*-wave without Γ-point hole pocket at SC I and *s*_±_-pairing at SC II^21^. In these systems, however, since no experimental study of not only the electronic structure, but also the crystal structure under pressure has been reported so far, the issue of the appearance of SC II dome under pressure remains unclear.

In this paper we report a systematic study of K_*x*_Fe_2−*y*_Se_2_ with x-ray diffraction (XRD) and x-ray emission spectroscopy (XES) under pressure. The purpose of this work is to reveal both the crystal and electronic structures of K_*x*_Fe_2−*y*_As_2_ under pressure to clarify the origin of the two superconducting domes. XES technique has made it possible to probe local magnetic moment under pressure by detecting Fe *Kβ* emission spectra for iron-based superconductor^22^,^23^,^24^,^25^,^26^. We also performed the bulk sensitive x-ray absorption (XAS) measurements with partial fluorescence (PFY) mode at the Fe *K* absorption edge^27^. We have used the PFY-XAS method where a decay process with shorter life time is selected, resulting spectra are narrower, and making fine electronic structure near the absorption edge better visible^27^,^28^,^29^. Our results show the change in the *c*-axis compressibility around boundary pressure of the SC I and SC II phases, suggesting a crystal structure change at this pressure, probably a tetragonal (T) to collapsed tetragonal (cT) transition. The Fe *Kβ* XES also shows a pressure-induced change in the electronic structure at the transition pressure.

## Results

### *P*-*T* phase diagram

**Figure 1:**
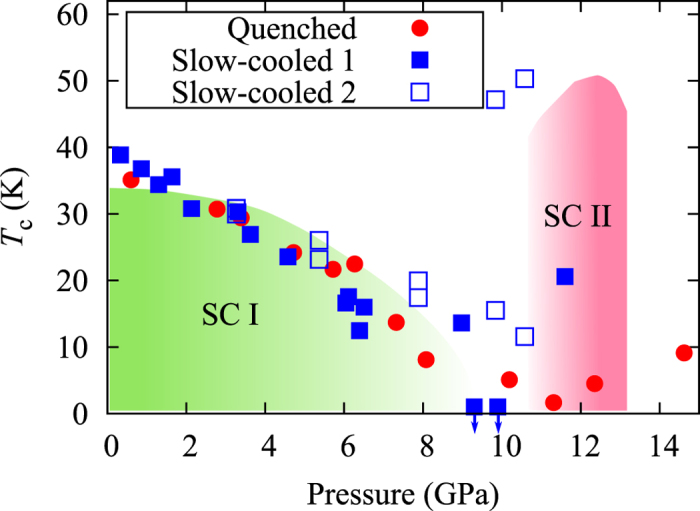
A *P*-*T* phase diagram of K_*x*_Fe_2−*y*_Se_2_^30^. Red circles and blue squares indicate quenched sample and slow-cooled sample, respectively. Colouring region is based on the data taken from the ref. 19.

We prepared two kinds of K_*x*_Fe_2−*y*_Se_2_ single crystals: a sample quenched at 550 °C (quenched sample) and one cooled slowly (slow-cooled sample). A *P*-*T* phase diagram of the quenched and slow-cooled samples is shown in Fig. 1. *T*_c_ was determined from the onset temperature of the electrical resistivity measurements. Both samples show that *T*_c_ decreases with pressure monotonically in the SC I phase. This behavior agrees well with the reports published^19^,^20^. However, the maximum *T*_c_ of SC II phase depends on the samples. *T*_c_ of the quenched and slow-cooled samples are ~5 K and ~20 K at the SC II phase, respectively^30^, while *T*_c_ of SC II was ~50 K in the reports published^19^,^20^. These results suggest that the *T*_c_ of SC II depends strongly on the sample preparation. Actually, island- and mesh-shape morphology were observed in the back-scattered electron (BSE) image in the slow-cooled and the quenched samples, respectively^31^. These morphologies were caused by the difference of iron concentration^31^.

### X-ray diffraction

**Figure 2:**
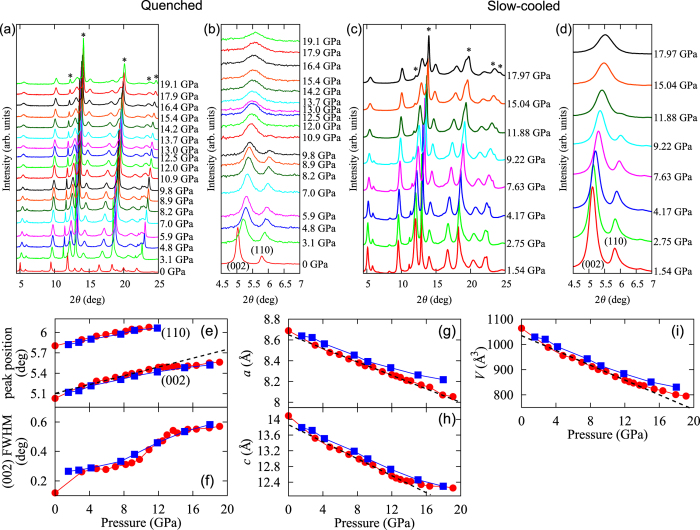
XRD pattern of (**a**) the quenched sample and (**c**) the slow-cooled sample. (**b,d**) Enlarged views of (**a,c**), respectively. Asterisk mark means reflection of NaCl used as the pressure medium of the diamond anvil cell. In the both quenched and slow-cooled samples, the (110) superstructure reflection disappear around 12 GPa. (**e–i**) Pressure evolution of the peak properties and the structure parameters of the quenched (red circle) and slow-cooled (blue square) samples. (**e**) Peak position of (002) and (110). (**f**) Full width at half maximum of the (002) peak. (**g**) Lattice constant along the *a*-axis. (**h**) Lattice constant along the *c*-axis. (**i**) Volume. Linear dashed-lines are guides for the eye.

We measured x-ray diffraction patterns under pressure up to 19.1 GPa for the quenched sample and 18.0 GPa for the slow-cooled sample at room temperature as shown in Fig. 2. Both samples consist of a *I*4/*m* symmetry of the 245 phase and a *I*4/*mmm* symmetry of the 122 phase at ambient pressure. Fe vacancy order-disorder transition was reported in the non-superconducting 245 phase at SC II, and crystal symmetry after the transition becomes *I*4/*mmm* which is the same as the superconducting phase^15^,^32^. Figure 2(a,c) show the XRD patterns of the quenched and slow-cooled samples, and the enlarge views are shown in Fig. 2(b,d). Intensity of the superstructure peak (110) attributed to the Fe vacancy order disappears around 12 GPa, indicating a clear structural phase transition from *I*4/*m* to *I*4/*mmm* symmetry at 245 phase. The same feature has been observed previously^15^,^32^. Seemingly, the above structural transition pressure of 12 GPa coincides with the appearance of the SC II phase as seen in Fig. 1.

Although a Rietveld refinement was not performed because of the restriction of the observed ***Q*** range, we performed peak fits by using the several peaks with the Voigt functions in order to derive the lattice constants. Figure 2(e) indicates (002) and (110) peak position vs pressure. Trend of the pressure evolution of (002) peak position changes around 12 GPa. This system consists of the 122 and 245 phases and thus only the average lattice constant of two phases could be analyzed. Since the 245 phase under pressure shows no superconductivity up to 22 GPa even though the cross over from the insulating phase to the metalic phase is found around 220 K at 0.4 GPa^20^, it is expected that only the 122 phase shows superconductivity in the whole SC region observed at ambient and high pressure. Therefore, it is likely that the volume fractions of the 122 phase should remain at 10–13% and 30–35% in the slow-cooled and quenched samples in the SC II region, respectively, (See Method section) Here, we assumed *I*4/*m* symmetry at all pressures because *I*4/*mmm* symmetry can express *I*4/*m* symmetry. Figure 2(g,i) show pressure evolution of the lattice constants. Pressure evolution of the *a*-axis shows a monotonic decrease, while that of the *c*-axis changes the slope around 12 GPa. Thus the compressibility along the *c*-axis becomes lower above 12 GPa. This means that the bond along the *c*-axis at the SC II phase is stronger than that at the SC I phase. This suggests a crystal structure change at 12 GPa, probably T → cT structural phase transition analogous to EuFe_2_As_2_^33^.

### Pressure induced change in the *Kβ* emission spectra

**Figure 3:**
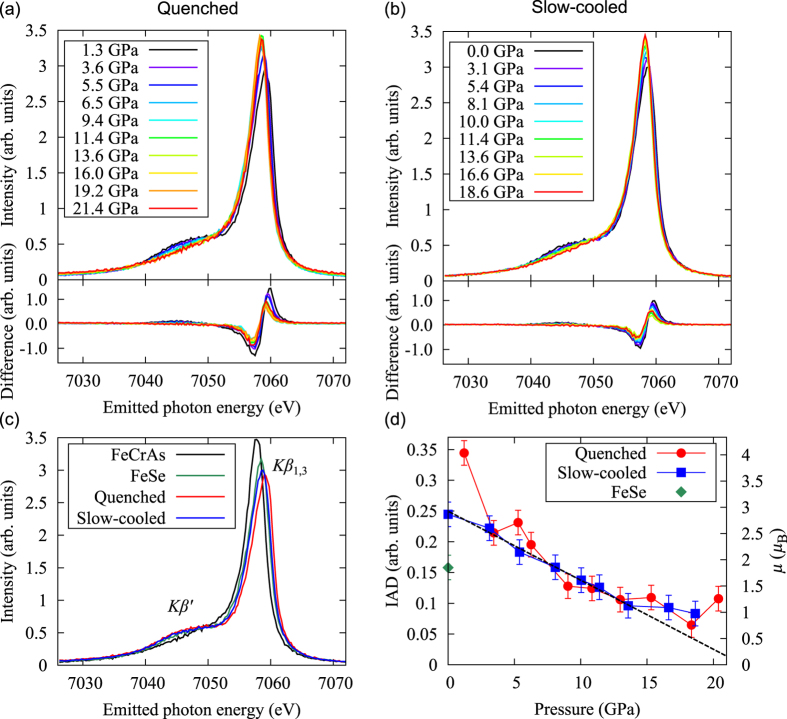
Pressure dependence of *Kβ* emission spectra of the (**a**) quenched and (**b**) slow-cooled samples. (**c**) *Kβ* spectra of FeCrAs, FeSe, the quenched sample, and the slow-cooled sample. (**d**) Pressure dependence of amplitude of magnetic moment per Fe estimated with the IAD values of the *Kβ* spectra. A linear dashed-line is a guide for the eye.

Figure 3(a,b) show pressure evolution of *Kβ* emission spectra of the quenched and slow-cooled samples, respectively. A *Kβ* spectrum consists of a main peak of *Kβ*_1,3_ and a satellite peak of *Kβ*′, which correspond to low-spin and high-spin states, respectively^22^. In Fig. 3, pressure evolution of *Kβ* spectrum shows a shift from the high-spin to the low-spin state with pressure.

Figure 3(c) shows a comparison among the *Kβ* spectra of the quenched sample, the slow-cooled sample, FeCrAs (0 *μ*_B_), and FeSe (2 *μ*_B_). As seen in Fig. 3(c), comparison of *Kβ* spectra between K_*x*_Fe_2−*y*_Se_2_ and FeCrAs concludes that K_*x*_Fe_2−*y*_Se_2_ is in the higher-spin state because of larger *Kβ*′ intensity. The local moment of Fe can be extracted by the the integrated absolute difference (IAD) analysis of the Fe *Kβ* emission spectra to a reference spectrum^23^,^25^. It is known that the IAD values are proportional to the local magnetic moments^25^.

Figure 3(d) shows the local magnetic moment estimated by the IAD analysis of the *Kβ* spectra in Fig. 3(a,b). The local magnetic moment decreases from ~3 *μ*_B_ at ambient pressure to ~1 *μ*_B_ at the SC II phase with pressure. Two samples show roughly the same trend under pressure. Especially the pressure evolution of the local magnetic moment of slow-cooled sample changes the slope at 12 GPa. This coincides with the change in the compressibility along the *c*-axis shown in Fig. 2(h).

### Pressure induced change in the PFY-XAS spectra

**Figure 4:**
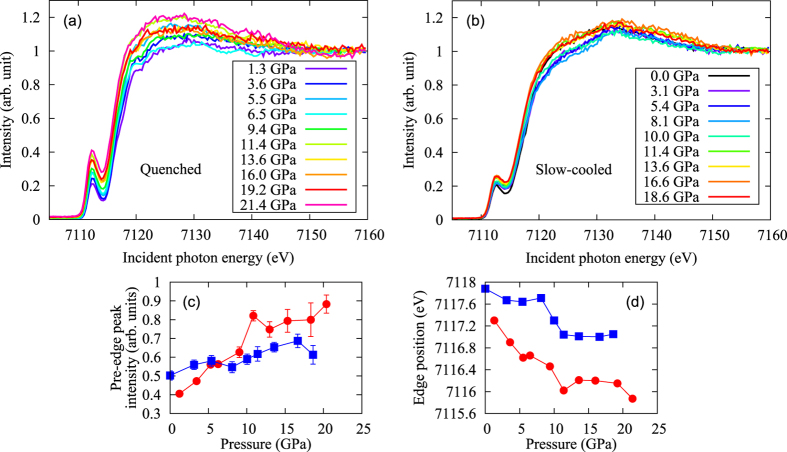
Pressure evolution of the PFY-XAS spectra of (**a**) the quenched sample and (**b**) the slow-cooled sample. In both the quenched and slow-cooled samples, the pre-edge peak intensity increase with pressure and edge position move towered to low energy. Pressure evolution of (**c**) the pre-edge peak intensity and (**d**) the edge position. Red circle and blue square indicate the quenched and slow-cooled samples, respectively.

Figure 4(a,b) show a pressure evolution of the PFY-XAS spectra setting the emitted photon energy to *Kβ*_1,3_ peak of the quenched and slow-cooled samples, respectively. The intensity is normalized to that at 7160 eV. The PFY-XAS spectra show large pre-edge peaks. The pre-edge and the main edge peaks correspond to 1*s* → 3*d* quadrupole and 1*s* → 4*p* dipole transitions, respectively. The strong pre-edge peak intensity includes the information of the hybridization between the Fe 3*d* and Se 4*p* orbitals^34^. The edge position of the PFY-XAS spectra shifts toward lower energy with pressure in both samples, indicating the decrease of the Fe valence. The system includes Fe^3+^ and Fe^2+^ 35 and thus the above result indicates a change in the Fe valence from Fe^3+^ → Fe^2+^. The decrease of the Fe valence with pressure may be due to the electron supply from K to FeSe layer caused by the shrink along the *c*-axis. Figure 4(c) shows that the pre-edge peak intensity of the PFY-XAS spectra increases with pressure. Another point we would like to emphasize is that the intensity around 7125 eV changes at 12 GPa in the slow-cooled sample, although it is not clear in the quenched one (Fig. 4(a,b)). This pressure also coincides with the pressure where the compressibility of the *c*-axis changes.

## Discussion

The XRD and XES studies under pressure have been performed for the 122* system, which have pressure-induced two superconducting domes. The XRD results show that the compressibility along the *c*-axis changes at 12 GPa and the superlattice diffraction disappears at the same pressure. Pressure dependence of the lattice constant along the *c*-axis and the volume becomes gentle at the SC II phase. The same *c*-axis evolution has been observed in *A*Fe_2_As_2_ where *A* = Ca, Sr, Ba and Eu^33^. This was interpreted as the T → cT structural phase transition. As shown in Fig. 2(f), the FWHM of the (002) peak in the XRD pattern starts to increase rather abruptly at around 12 GPa. This can be explained if we make the assumption that the (002) peak consists of two components. One peak (Supplementary Fig. 2) appears to originate from the 245 phase, which remains structurally stable up to 22 GPa, by considering previous high-pressure resistivity data^20^, and the other from the 122 phase, which undergoes a cT phase transition. We note that the quenched sample shows a more sudden change than that of the slow-cooled sample. This may be related to the SC volume fraction difference between them. Since the 122 phase in K_*x*_Fe_2−*y*_Se_2_ has the same crystal structure as that of *A*Fe_2_As_2_ (*A* = Ca, Sr, Ba, Eu), it is reasonable to expect that the 122 phase in K_*x*_Fe_2−*y*_Se_2_ shows the same phase transition as *A*Fe_2_As_2_ under pressure. This coexistence of two phases is likely to lead to a smearing out of the T to cT phase transition, as compared, for example, to the clear discontinuity observed in EuFe_2_As_2_^33^. The change in the crystal structure affects the magnetic property. Actually, the *Kβ* XES results indicate that the trend of the pressure evolution of the magnetic moment and the electronic state shown in Fig. 3(d) changes also at 12 GPa, which seems to correlate to the T → cT transition. The average local magnetic moment changes from ~3 *μ*_B_ at ambient pressure to ~1 *μ*_B_ at the SC II phase with pressure. The change in the magnetic moment at 12 GPa is not large in K_*x*_Fe_2−*y*_Se_2_ system, probably because the collapse along the *c*-axis at 12 GPa is small.

The PFY-XAS spectra show that the Fe valence decreases with pressure, which may correspond to the increase of the carrier density at the SC II phase due to the supply of the electrons from K to the FeSe layer caused by the shrink along the *c*-axis. The pre-edge peak intensity in the PFY-XAS spectra increases with pressure, indicating the increase of the hybridization between Fe 3*d* and Se 4*p* and also the density of states (DOS) near the Fermi surface. The pressure-induced change in the pre-edge peak intensity also correlates to the shift from high-spin to low-spin states (See also Supplementary Fig. 5).

In the 122 system the superconductivity emerged suddenly at the cT phase when the T → cT structural phase transition occurred^36^. The phase diagram of the 122 system is similar to that of the 122* system^37^. The DFT calculations showed the change in the electronic structure between the T phase and cT phase^38^. In KFe_2_As_2_, the T → cT transition changed the superconductivity symmetry from *d*-wave to *s*-wave. This is a Lifshitz transition which is known to change the Fermi surface drastically from the electronic state with only hole pocket to that with electron and hole pockets. Other calculations of K_*x*_Fe_2−*y*_Se_2_ also showed the *d*-wave in the SC I and *s*-wave in the SC II phase^21^. Therefore, together with these theoretical calculations we conclude that K_*x*_Fe_2−*y*_Se_2_ shows the T → cT transition and the increase of the density of states near the Fermi surface under pressure, which are the key evidence for understanding the appearance of the SC II phase.

## Methods

### Sample preparation and characterizations

We prepared two kinds of K_*x*_Fe_2−*y*_Se_2_ single crystals^31^,^39^. Single crystals were grown by a simple one-step synthesis. Fe (99.9%), K_2_Se (99%) powders and Se (99.999%) grains were put into an alumina crucible and sealed into an evacuated quartz tube. The mixture was slowly heated up to 900 °C and held for 3 hours. The melting mixture was, then, cooled down to room temperature slowly (slow-cooled sample) and quenched at 550 °C (quenched sample). Back-scattered electrons (BSE) images were obtained to observe micro-structure. Island- and mesh-shape structure were shown in the slow-cooled and quenched samples, and the chemical composition determined by using energy dispersive x-ray spectrometry (EDX) were K_0.40_Fe_1.95_Se_2_ and K_0.63_Fe_1.71_Se_2_, respectively^31^. The area ratios between the superconducting region and non-superconducting region is ~10–13% in the slow-cooled sample and ~30–35% in the quenched sample. *T*_c_ of the present samples under pressure were measured at Osaka University^30^.

### XRD, XES, and PFY-XAS measurements under pressure

We performed XRD, XES, and PFY-XAS experiments for the slow-cooled and quenched samples. For XRD, XES, and PFY-XAS measurement, these samples with NaCl as the pressure medium were loaded into a sample chamber of the gasket in the glove box of pure Ar atmosphere because these samples are chemically unstable in the air. Pressure was monitored by ruby fluorescence method^40^.

Pressure dependence of the XRD patterns were measured at SPring-8 BL12B2 using a 3-pin plate diamond anvil cell (DAC, Almax Industries) with a CCD detection system at room temperature. We took an arrangement of both incoming and outgoing x-ray beams passed through the diamonds with incident photon energy of 20 keV. NaCl was loaded as the pressure medium and well-mixed with the sample because of reduction of preferred orientation of the sample. (Supplementary Fig. 1) 2D image of CCD was integrated by using FIT2D program^41^.

The PFY-XAS and XES measurements were performed at the Taiwan beam line BL12XU at SPring-8. The undulator beam was monochromatized by a cryogenically-cooled double crystal Si(111) monochromator. A Johann-type spectrometer equipped with a spherically bent Si(531) analyzer crystal (radius of ~1 m) and a Si solid state detector (Amptech) were used to analyze the Fe emission of the 3*p* → 1*s* de-excitation at the Fe *K* absorption edge. At the emitted photon energy of 7.6 keV the overall energy resolution was estimated to be 0.9 eV. The intensities of the measured spectra were normalized using the incident beam that was monitored just before the sample.

For the high-pressure XES experiments the x-ray beam was focused to 20–30 (horizontal) × 30–40 (vertical) *μ*m^2^ at the sample position using a toroidal and a Kirkpatrick-Baez mirror. High-pressure conditions were achieved at room temperature using a diamond anvil cell coupled with a gas-membrane. A Be-gasket with 3 mm in diameter and approximately 100 *μ*m thick was pre-indented to approximately 35–40 *μ*m thickness around the center. The diameter of the sample chamber in the gasket was approximately 100 *μ*m and the diamond anvil culet size was 300 *μ*m. We used the Be gasket in-plane geometry with a scattering angle of 90°, where both incoming and outgoing x-ray beams passed through the Be gasket. Be was used due to its higher transmittance to x-rays in comparison to other high-*Z* materials.

### IAD analyses

The IAD analysis is performed in the following way: (i) match the center of mass between the sample and reference spectra, (ii) take the difference between them, and (iii) integrate the absolute value of the difference. The intensity is normalized by the area of the *Kβ* spectrum.

## Additional Information

**How to cite this article**: Yamamoto, Y. *et al*. Origin of Pressure-induced Superconducting Phase in K*_x_*Fe_2−*y*_Se_2_ Studied by Synchrotron X-ray Diffraction and Spectroscopy. *Sci. Rep.* **6**, 30946; doi: 10.1038/srep30946 (2016).

## Supplementary Material

Supplementary Information
