# Supplementary material for: Origin of Pressure-induced Superconducting Phase in KxFe2−ySe2 studied by Synchrotron X-ray Diffraction and Spectroscopy
Source: Sci Rep. 2016 Aug 8;6:30946. doi: 10.1038/srep30946 (PMC4976351; doi:10.1038/srep30946)
Supplement: Supplementary Information [file srep30946-s1.pdf]

Supplemental information: Origin of Pressure-induced Superconducting Phase in $K_xFe_{2-y}Se_2$ studied by Synchrotron X-ray Diffraction and Spectroscopy

Yoshiya Yamamoto,[1] Hitoshi Yamaoka,[2] Masashi Tanaka,[3] Hiroyuki Okazaki,[3,4] Toshinori Ozaki,[3,1] Yoshihiko Takano,[3] Jung-Fu Lin,[5,6] Hidenori Fujita,[7] Tomoko Kagayama,[7] Katsuya Shimizu,[7] Nozomu Hiraoka,[8] Hirofumi Ishii,[8] Yen-Fa Liao,[8] Ku-Ding Tsuei,[8] and Jun'ichiro Mizuki[1]

[1]*Graduate School of Science and Technology, Kwansei Gakuin University, 2-1 Gakuen, Sanda, Hyogo 669-1337, Japan*
[2]*RIKEN SPring-8 Center, RIKEN, 1-1-1 Kouto, Mikazuki, Sayo, Hyogo 679-5148, Japan*
[3]*MANA, National Institute for Materials Science, 1-2-1 Sengen, Tsukuba, Ibaraki 305-0047, Japan*
[4]*Advanced Institute for Materials Research, Tohoku University, 2-1-1 Katahira, Aoba, Sendai, Miyagi 980-8577, Japan*
[5]*Department of Geological Sciences, The University of Texas at Austin, Austin, Texas 78712, USA*
[6]*Center for High Pressure Science and Technology Advanced Research (HPSTAR), Shanghai 201203, China*
[7]*Center for Science and Technology under Extreme Conditions (KYOKUGEN), Graduate School of Engineering Science, Osaka University, Toyonaka, Osaka 560-8531, Japan*
[8]*National Synchrotron Radiation Research Center, Hsinchu 30076, Taiwan*

Here we show additional information about the results of the XRD and the PFY-XAS and a correlation of the magnetic moment with T_c.

X-RAY DIFFRACTION

Figure 1 shows an example of the 2D XRD pattern. There is no spot peak with preferred orientation. Note that this image includes the strong peak from diamond. We integrated the image with masking by using FIT2D program.[1]

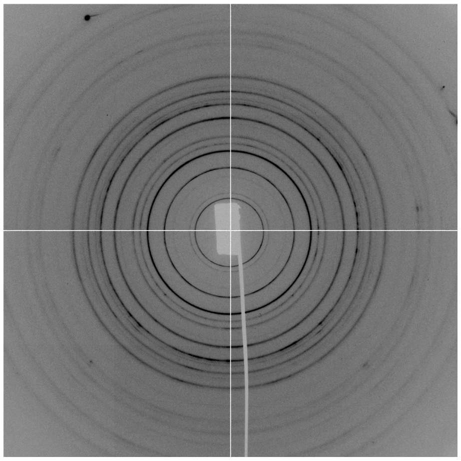

FIG. 1. (Color online) An example of the 2D XRD pattern of the quenched sample.

Figure 2 shows full width at half maximum (FWHM) of the (200) peak of NaCl used as the pressure medium of the diamond anvil cell. Change in the FWHM of (200) peak increases smoothly with pressure and no sudden change is observed in the pressure range measured. Therefore, the trend of the pressure-induced change in the lattice constant along the c-axis around 12 GPa is reliable.

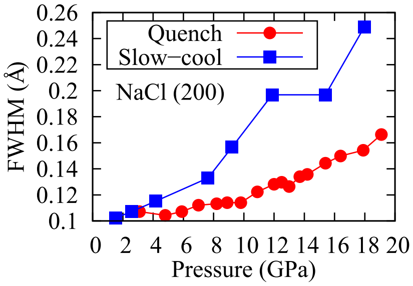

FIG. 2. (Color online) Full width at half maximum (FWHM) of the (200) peak of NaCl used as the pressure medium of the diamond anvil cell. Red closed circle and blue closed square correspond to the quenched and slow-cooled samples, respectively.

Figure 3 shows the anion height and the As-Fe-As bond angle estimated from the lattice constants. In $EuFe_2As_2$, Wyckoff positions change with pressure, especially z position changes dynamically.[2] However, roughly, the bond angle takes an optimum value of $\sim$109° at the SC II phase and the anion height does an optimum value of $\sim$1.38 Å at the SC I phase.[3] The bond angle increases with pressure, while the anion height decreases monotonically. Both the trend of the changes in the bond angle and anion height seems to be gentle above 12 GPa.

PFY-XAS

Figure 4 shows PFY-XAS spectra of FeCrAs ($0\mu_B$), FeSe ($2\mu_B$) and $K_xFe_{2-y}Se_2$ ($3.3\mu_B$). We measured two kinds of the PFY-XAS spectra by setting the emitted photon energies to the $K\beta_{1,3}$ and $K\beta'$ peaks, respectively. The intensity of the pre-edge peak of the PFY-XAS spectra at the $K\beta'$ peak (red lines in Fig. 3) decreases with increasing

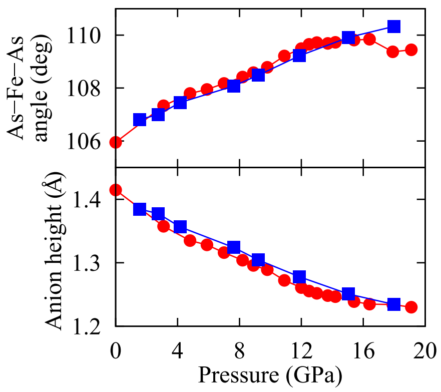

FIG. 3. (Color online) Anion height and As-Fe-As bond angle calculated from lattice constants of the quenched samples (closed square) and slow-cooled sample (closed circle).

magnetic moment. Both the PFY-XAS spectra show same feature above the K-absorption energy. It is considered that the difference of the feature of the pre-edge peak reflects the spin state. The intensity of the pre-edge peak of the spectra at the $K\beta'$ peak is weak normally in the high-spin state.[4] Figure 4 shows higher-magnetic moment correlates to the lower intensity of the pre-edge peak of the PFY-XAS spectra at the $K\beta'$ peak, reasonably corresponding to the higher-spin state.

The pre-edge peak intensity at the $K\beta_{1,3}$ increases with pressure, corresponding to the change in the spin state from the high-spin to low-spin state. This also suggests that Fe 3d-Se 4p hybridization becomes stronger with pressure and thus the DOS near the Fermi surface is higher at the high pressure phase.

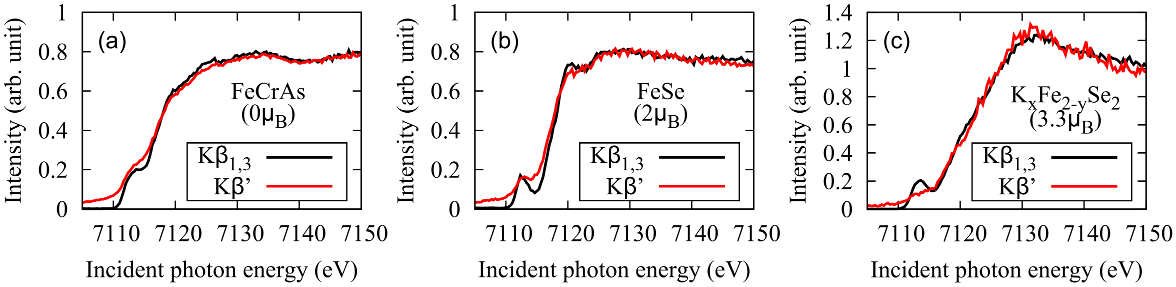

FIG. 4. (Color online) PFY-XAS spectra of (a) FeCrAs ($0\mu_B$) and (b) FeSe ($2\mu_B$) and (c) $K_xFe_{2-y}Se_2$ ($3.3\mu_B$) set the emitted photon energy to the $K\beta_{1,3}$ peak (red line) and $K\beta'$ peak (blue line).

Figure 5 shows the pressure dependence of the PFY-XAS spectra. In Figs. 5(c) and 5(d) we show the pressure dependence of the differential of the PFY-XAS spectra near the pre-edge peak region. The pressure-induced change in the peak position of the differential of the PFY-XAS spectra indicates that the inflection point changes to lower energy with pressure as shown in Fig. 5(e). The evolution of the inflection point corresponds to that of the chemical potential.[5] Thus the chemical potential decreases with pressure at SC II phase. Bendele *et al.* showed that the chemical potential at the SC II phase was lower than that at the SC I phase, suggesting the increase of the density of states with pressure.[5] Our results show a similar trend except 7-11 GPa, where a gradual change in the chemical potential is suggested. The peak positions gradually drop with pressure above 5 GPa at the SC I phase and do not change much at the SC II phase above 10 GPa.

Figure 6 shows a correlation of the magnetic moment μ and T_c at the SC I phase. The data of T_c in the p-T phase diagram was fitted and T_c at a given pressure was estimated. There is an apparent correlation between the magnetic moment and T_c. Theoretically, it was suggested that a spin-lattice coupling possibly occurs in the Fe pnictide superconductors and the Fe local moment correlates to the Fe-As layer separation, i. e. the anion height.[6] The anion height as well as T_c decreases with pressure at the SC I phase as shown in Fig. 3. Thus, the above theory can explain that the pressure-induced change in the local magnetic moment derived from the $K\beta$ XES spectra reasonably correlates to the superconductivity at the SC I phase. But further theoretical and experimental studies will be required to understand the correlation between the superconductivity and local magnetism.

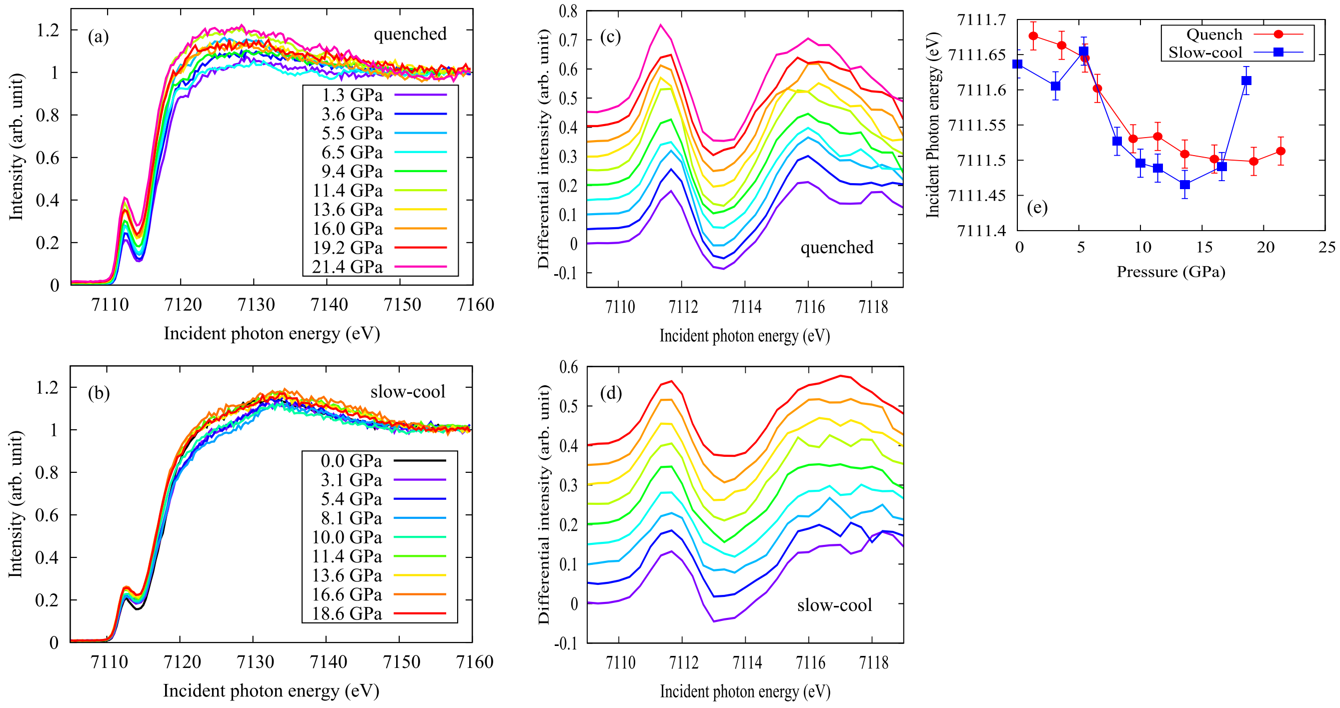

FIG. 5. (Color online) Pressure evolution of the PFY-XAS spectra of (a) the quenched and (b) the slow-cooled samples. In both the quenched and slow-cooled samples, the pre-edge peak intensity increases with pressure and the edge position moves towered the low energy. (c) and (d) Pressure evolution of the differential of the PFY-XAS spectra around the pre-edge peak. Upper and lower spectra correspond to high and low pressures, respectively. (e) Pressure evolution of the peak position of the differential PFY-XAS spectra around 7111.5 eV.

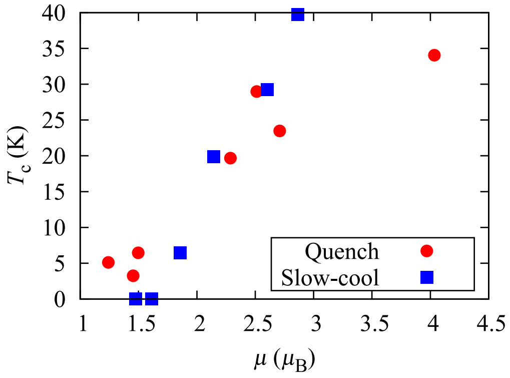

FIG. 6. (Color online) Correlation between μ and T_c at the SC I phase.

[1] Hammersley, A. P., Svensson, S. O., Hanfland, M., A. N. Fitc, h. & Hausermann, D. Two-dimensional detector software: From real detector to idealised image or two-theta scan. *High Pressure Research* **14**, 235; DOI:10.1080/08957959608201408 (1996).

[2] Yu, Z. *et al.* Conventional empirical law reverses in the phase transitions of 122-type iron-based superconductors. *Sci. Rep.* **4**, 7172; DOI:10.1038/srep07172 (2014).

[3] Mizuguchi, Y. *et al.* Anion height dependence of T_c for the Fe-based superconductor. *Supercond. Sci. Technol.* **23**, 054013; DOI:10.1088/0953-2048/23/5/054013 (2010).

[4] Yamaoka, H. *et al.* $K\beta$ resonant x-ray emission spectroscopy for Fe, Fe_2O_3 and Fe_3O_4. *Journal of the Physical Society of Japan* **73**, 3182–3191; DOI:10.1143/JPSJ.73.3182 (2004).

[5] Bendele, M. *et al.* Interplay of electronic and lattice degrees of freedom in $A_{1-x}Fe_{2-y}Se_2$ superconductors under pressure.

Phys. Rev. B **88**, 180506; DOI:10.1103/PhysRevB.88.180506 (2013).
[6] Egami, T., Fine, B. V., Parshall, D., Subedi, A. & Singh, D. J. Spin-lattice coulping and superconductivity in Fe pnictides. *Adv. Condens. Matter. Phys.* **2010**, 164916; DOI:10.1155/2010/164916 (2010).
